# Supplementary material for: Complexome profiling of the Chlamydomonas psb28 mutant reveals TEF5 as an early PSII assembly factor
Source: Plant Cell. 2025 Mar 17;37(6):koaf055. doi: 10.1093/plcell/koaf055 (PMC12164590; doi:10.1093/plcell/koaf055)
Supplement: koaf055_Supplementary_Data [file koaf055_supplementary_data.zip › psb28_interactive.zip!/plots/intensity/qualityPlots/peptides/LCI11B_Cre16.g663450.t1.2.html]

### Cre16.g663450.t1.2

Trivial name: LCI11B  
SeqCoverage [median]: 9.70  
Mass: 50.60 kDa  
  
 

| Sequence | MissedCleavage | Mass [Da] | wtIntensity | muIntensity | Score |
| --- | --- | --- | --- | --- | --- |
| AIHAISQIIQSVR | 0 | 1434.83 | 496.10 | 930.19 | 56.95 |
|  |  |  | 496.1 | 930.2 |  |
